# The Virtual Fracture Clinic Bottleneck: A Single-Centre Service Evaluation of Compliance With British Orthopaedic Association Surgical Timing Standards for Distal Radius Fixation

**DOI:** 10.7759/cureus.114475

**Published:** 2026-08-13

**Authors:** Ahmed Mohammed, Adithya Venkatasubramanian

**Affiliations:** 1 Trauma and Orthopaedics, Lincoln County Hospital, Lincoln, GBR; 2 Trauma and Orthopaedics, United Lincolnshire Teaching Hospitals NHS Trust, Lincoln, GBR

**Keywords:** boast guidelines, clinical audit, distal radius fracture, surgical timing, trauma triage, virtual fracture clinic

## Abstract

Background: Distal radius fractures are common trauma presentations. The British Orthopaedic Association Standards for Trauma (BOAST) guidelines mandate surgical fixation within 72 hours for unstable intra-articular fractures and within seven days for extra-articular fractures. Virtual fracture clinics (VFCs) are now the dominant National Health Service (NHS) triage model, but their effect on time-critical surgical decision-making is unclear. This service evaluation assessed whether the VFC pathway delays operative management of distal radius fractures relative to BOAST standards, compared with traditional trauma-meeting triage.

Methods: We conducted a retrospective service evaluation of adult patients undergoing open reduction and internal fixation (ORIF) of a distal radius fracture over seven months at Lincoln County Hospital, United Lincolnshire Teaching Hospitals NHS Trust, Lincoln, England, United Kingdom. Patients were stratified by triage route (VFC vs. traditional trauma meeting) and fracture morphology. BOAST compliance was compared between pathways using Fisher's exact test, with odds ratios (ORs) and 95% confidence intervals (CIs).

Results: Of 25 ORIF cases, 92% (23/25) were intra-articular. Overall BOAST compliance was 12% (3/25). Compliance was 0% (0/17) in the VFC cohort versus 37.5% (3/8) in the traditional trauma-meeting cohort (Fisher's exact p = 0.0243; OR 0.045, 95% CI 0.002-0.93).

Conclusion: The VFC pathway was associated with significantly reduced BOAST-compliant surgical timing. This association may in part reflect an additional clinic step prior to listing, although causation cannot be established from this retrospective design; the findings should be regarded as hypothesis-generating. Departments using VFC pathways should consider a direct-listing escalation protocol for fractures meeting operative criteria, to avoid breaching hyper-acute BOAST windows.

## Introduction

Distal radius fractures are among the most common injuries presenting to trauma services, and management decisions are guided principally by fracture morphology and radiographic assessment [1,2]. The overarching aim of treatment is restoration of functional wrist mobility rather than strict adherence to radiographic parameters alone [3]. Where operative fixation is indicated, the British Orthopaedic Association Standards for Trauma (BOAST) guidelines mandate that unstable intra-articular fractures undergo surgical stabilisation within 72 hours of injury, extra-articular fractures within seven days, and re-displaced fractures within 72 hours of the decision to operate [1,2].

Distal radius fractures also represent a substantial burden on National Health Service (NHS) trauma services: a 10-year analysis of Hospital Episode Statistics data identified them as the second most common fracture requiring hospital admission in England, accounting for 17% of all fracture admissions (406,313 cases) between 2004 and 2014, second only to hip fractures [4]. Efficient and equitable triage of this high-volume injury is therefore of considerable service-level importance, particularly for the minority of patients who meet criteria for operative fixation and are consequently subject to the time-critical BOAST standards described above.

The rationale for these hyper-acute timing thresholds lies principally in the biomechanics of fracture healing and soft-tissue compliance. Intra-articular fractures are prone to progressive fracture-fragment settling and increasing peri-fracture soft-tissue swelling in the days following injury, both of which can compromise closed reduction and necessitate more extensive surgical exposure if fixation is delayed. A recent cohort analysis quantified this relationship directly, demonstrating that radiographic alignment deteriorates linearly with each additional day of delay to surgery, with the risk of an unacceptable final reduction approximately doubling after a two-week delay [5]. Prompt surgical stabilisation is therefore intended not merely as a process target but as a means of preserving the surgeon's ability to achieve an anatomical reduction using a minimally invasive technique.

Virtual fracture clinics (VFCs) were introduced across the NHS following the 2013 BOAST 7 standard, which required new fracture-clinic review within 72 hours of injury, and have since become the dominant triage model for acute orthopaedic trauma in the UK [6]. VFCs allow senior clinicians to remotely review radiographs and clinical information asynchronously, triaging patients to discharge, physiotherapy, or face-to-face review without requiring in-person attendance. Published series report substantial improvements in BOAST-7 clinic-review compliance following VFC implementation - in one case from 6% to 100% - and cost savings compared with traditional outpatient models [7,8,9]. However, the majority of this evidence addresses time-to-clinic-review rather than time-to-surgery, and a 2020 systematic review noted limited data specifically on operative timeliness within VFC models [10]. It therefore remains unclear whether the additional administrative review step inherent to the VFC model delays operative decision-making for the minority of patients who require urgent surgical fixation.

This service evaluation aimed to determine whether the administrative architecture of the VFC pathway introduces delays that compromise compliance with BOAST surgical timing standards for distal radius fractures, particularly unstable intra-articular fractures requiring fixation within the mandatory 72-hour window, compared with patients triaged via the traditional face-to-face trauma meeting.

## Materials and methods

Study design and population

This retrospective clinical service evaluation was conducted over a seven-month period at Lincoln County Hospital, United Lincolnshire Teaching Hospitals NHS Trust, Lincoln, England, United Kingdom. The project was registered with the trust's Clinical Governance and Audit Department (Registration No. L1446). As the evaluation used exclusively anonymised, routinely collected data, it was classified as a service evaluation rather than research; in accordance with NHS Health Research Authority guidance, formal ethical review was not required.

All adult patients who underwent operative management of a distal radius fracture by open reduction and internal fixation (ORIF) within the study period were included. Patients managed conservatively, or with closed reduction and cast immobilisation alone without surgery, were excluded.

Data collection

Patient data were extracted from electronic hospital records and theatre logbooks. Variables recorded included baseline demographics, fracture morphology (classified radiographically as intra-articular or extra-articular), the date and time of injury, the date of initial clinical triage, and the date of definitive surgical intervention. Data were entered into a structured Microsoft Excel collection sheet and used to calculate the time interval from injury to surgery for each case.

Pathway cohorts

Patients were stratified into two triage cohorts according to the route by which surgical listing was achieved: the VFC pathway, comprising patients whose initial emergency department presentation was uploaded electronically for asynchronous consultant review the following day, and the traditional trauma-meeting pathway, comprising patients discussed directly at the face-to-face daily trauma meeting, bypassing remote triage and enabling immediate surgical listing.

Allocation between the two pathways was not governed by a standardised or randomised clinical protocol, and the specific basis on which individual patients were routed to one pathway rather than the other could not be reliably reconstructed from the retrospective records available. This absence of a defined, auditable allocation process is itself a limitation of the pathway as delivered, rather than an incidental gap in this evaluation, and means that confounding by injury severity, referring clinician judgement, or other unmeasured factors between the two cohorts cannot be excluded (see Limitations). Other operational factors, including theatre list availability, on-call consultant workload, and patient-specific considerations, such as medical optimisation, may also have influenced the timing of definitive surgery once a patient had been listed, independent of triage route.

Statistical analysis

Categorical outcomes (BOAST-compliant vs. non-compliant surgical timing) were compared between triage pathways (VFC vs. traditional trauma meeting) using a two-tailed Fisher's exact test, given the small sample size (N = 25); statistical significance was defined as p < 0.05. An odds ratio (OR) with 95% confidence interval (CI) was calculated to quantify the relative odds of achieving BOAST-compliant surgical timing between the two pathways, applying a Haldane-Anscombe continuity correction owing to a zero-value cell in the contingency table. Fisher's exact test was selected in preference to the chi-squared test because expected cell counts were below five in more than one cell of the 2 × 2 contingency table, violating the assumptions required for a valid chi-squared approximation given the small overall sample size. A two-tailed p-value of less than 0.05 was considered statistically significant throughout.

## Results

A total of 25 distal radius ORIF cases met the inclusion criteria during the seven-month study period. Radiographic analysis classified 92% (23/25) of cases as intra-articular fractures and 8% (2/25) as extra-articular fractures.

**Figure 1:**
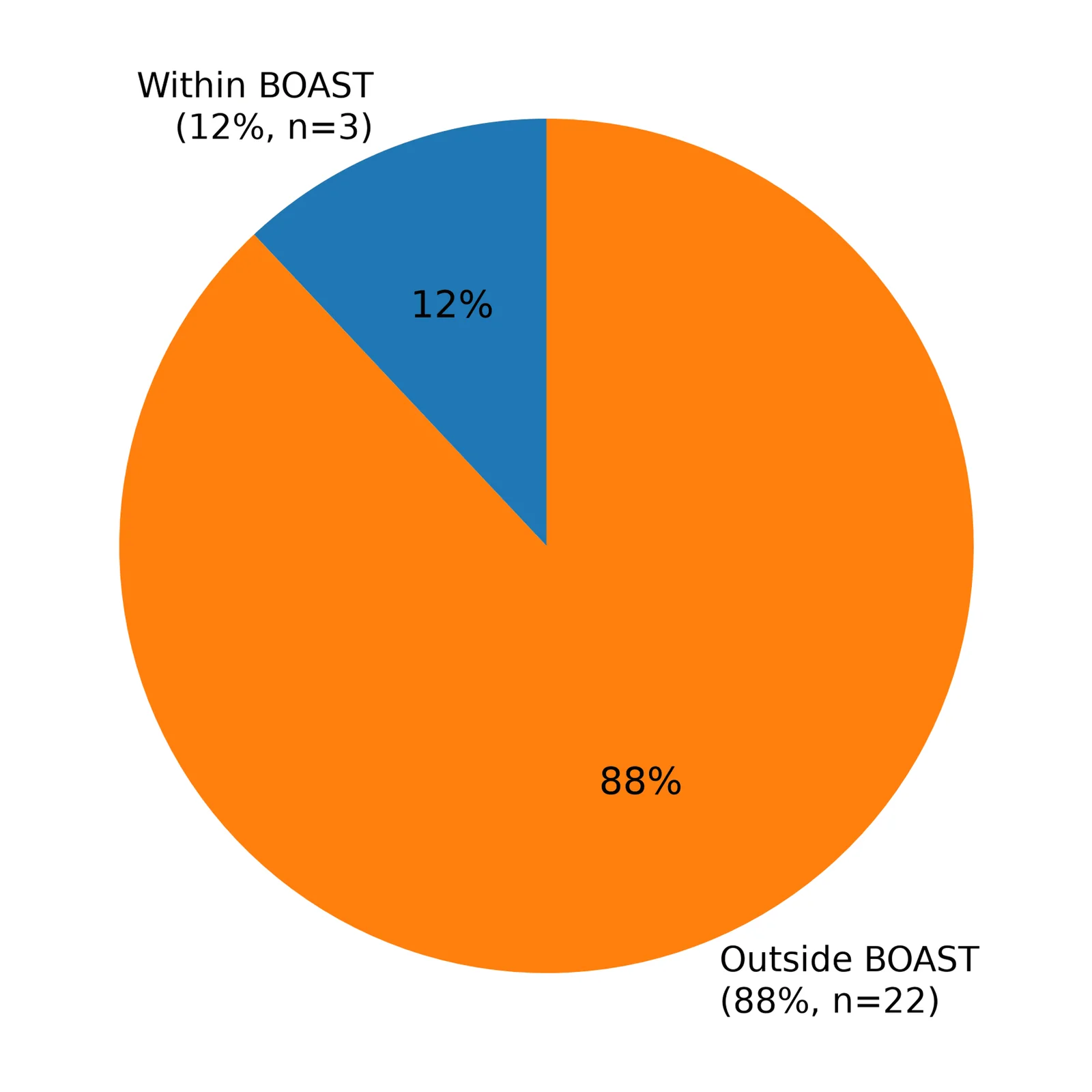
Overall compliance with BOAST surgical timing standards (n = 25). Pie chart showing the proportion of distal radius ORIF cases meeting BOAST-mandated surgical timing thresholds (12% compliant, 3/25) versus non-compliant (88%, 22/25). BOAST, British Orthopaedic Association Standards for Trauma

Overall departmental compliance with BOAST surgical timing guidelines was low, at 12% (3/25) (Figure 1). When stratified by fracture type, 100% (2/2) of extra-articular fractures were operated on within the seven-day target, whereas only 4.3% (1/23) of intra-articular fractures achieved definitive surgical stabilisation within the required 72-hour window.

When stratified by triage route, 17 patients were processed via the VFC pathway and eight via the traditional trauma-meeting pathway. Compliance with BOAST surgical timing was 0% (0/17) in the VFC cohort, compared with 37.5% (3/8) in the traditional trauma-meeting cohort (Table 1). Fisher's exact test confirmed that this difference was statistically significant (p = 0.0243), with an odds ratio of 0.045 (95% CI: 0.002-0.93) indicating substantially reduced odds of BOAST-compliant surgical timing when patients were routed through the VFC pathway. This corresponds to all 17 VFC-pathway patients experiencing a delay beyond the BOAST-mandated timing window, irrespective of fracture morphology.

**Table 1:** Compliance with BOAST surgical timing guidelines, stratified by the triage pathway. BOAST, British Orthopaedic Association Standards for Trauma; VFC, virtual fracture clinic

Pathway	Compliant	Non-compliant	Total	% Compliance
Virtual fracture clinic (VFC)	0	17	17	0%
Traditional trauma meeting	3	5	8	37.5%
Total	3	22	25	12%

The magnitude of this odds ratio should be interpreted with caution. Because zero VFC-pathway patients achieved BOAST-compliant timing, the 2 × 2 contingency table contained a zero-value cell, and the reported OR of 0.045 was calculated only after applying a Haldane-Anscombe continuity correction (adding 0.5 to each cell); without this correction, the OR would be mathematically undefined. The correspondingly wide 95% CI (0.002-0.93) reflects the small sample size and sparse-data nature of the comparison, and the point estimate itself should be regarded as indicative of a strong directional association - essentially, the complete absence of compliant cases in the VFC cohort - rather than as a precise measure of effect size.

Review of the audit dataset also identified a difference in the process pathway between the two cohorts that had not been prospectively captured as a discrete study variable: 94% (16/17) of VFC-pathway patients had a documented face-to-face clinic review prior to surgical listing, compared with 0% (0/8) of traditional trauma-meeting patients. This provides direct, data-based support - rather than a purely case-note-based inference - for the presence of an additional clinic step specific to the VFC pathway, although it does not by itself establish that this step caused the observed timing delay.

## Discussion

Principal findings

The principal finding of this service evaluation is that the administrative architecture of the VFC model is associated with a significantly reduced likelihood of meeting hyper-acute BOAST surgical timing targets for distal radius fractures. While VFC structures have demonstrated clear benefits for outpatient efficiency and clinic-review compliance elsewhere in the literature [7,8,9], our data suggest they may be poorly suited to the time-critical decision-making required for unstable intra-articular injuries. A 0% compliance rate across an entire 17-patient cohort, against 37.5% in the comparator pathway, represents a striking absolute difference that is unlikely to be explained by chance alone. This association should not, however, be interpreted as evidence of a causal relationship: the retrospective, non-randomised design means that structural features of the VFC pathway and unmeasured confounders (see Limitations) cannot be disentangled from these data alone, and the finding is best regarded as hypothesis-generating rather than confirmatory.

A hypothesised mechanism for the pathway bottleneck

Unlike the overall timing comparison, the presence of an additional clinic step within the VFC pathway is directly supported by our data rather than purely inferred: 94% (16/17) of VFC-pathway patients had a documented face-to-face clinic review before surgical listing, compared with 0% (0/8) of traditional trauma-meeting patients (see Results). This confirms that an additional administrative and clinical step is systematically present in the VFC pathway and absent from the comparator pathway. What remains hypothesis-generating, because granular timestamps for each administrative step (electronic upload, remote consultant review, and clinic booking) were not systematically captured as discrete variables in this evaluation, and no formal process-mapping exercise was undertaken, is the precise causal contribution of this extra step to the observed timing delay, as opposed to other unmeasured factors. We propose the following as a plausible explanatory model, informed by the clinic-review finding above together with informal case-note review, that would benefit from prospective verification with timestamped data. A patient presenting to the emergency department on Day 0 may be uploaded to the virtual triage pool on Day 1, undergo asynchronous remote consultant review on Day 2, and, rather than proceeding directly to a theatre list, be scheduled for an in-person outpatient clinic appointment to discuss operative management, with clerical processing and surgical booking potentially delayed to Day 3 or later. If this sequence is representative, interposing a face-to-face clinic assessment after remote review would leave little margin within the strict 72-hour BOAST window for intra-articular fractures, in contrast to the traditional trauma-meeting pathway, which allows same-day discussion and direct listing. The precise contribution of this mechanism to the timing delay remains unconfirmed and should be tested directly with timestamped process data in future work.

Comparison with existing literature

Our findings should be interpreted alongside the existing VFC literature, which is largely favourable. A widely cited service evaluation reported that VFC implementation improved BOAST-7 clinic-review compliance from 6% to 100% [5], and a 2025 review reported VFC compliance rates for specialist review consistently above 83%, compared with rates as low as 5.7% in some traditional clinics [6]. These studies, however, measure time to clinic review rather than time to surgery, and a 2020 systematic review explicitly identified a lack of evidence regarding the effect of VFC triage on operative timeliness [10]. Published national audits of traditional (non-VFC) pathways have themselves reported intra-articular compliance rates in the range of 33-65% [11], suggesting that our observed 4.3% intra-articular compliance rate is markedly lower than would be expected from case complexity alone. Taken together with the direct evidence of an additional clinic step in 94% of VFC-pathway patients, this pattern is consistent with the VFC administrative step being an associated contributory factor, although a causal relationship cannot be confirmed from this observational, unadjusted comparison.

Clinical significance of surgical delay

The clinical consequences of the timing breaches identified here warrant cautious interpretation. Not all evidence indicates that delayed fixation of distal radius fractures translates into measurably worse patient outcomes: a comparative study of patients undergoing surgery within versus beyond BOAST-recommended windows found no statistically or clinically significant differences in outcome measures between timely and delayed cohorts [12]. This raises the possibility that BOAST timing standards, while a valid process measure of departmental efficiency, may not map directly onto patient-reported or functional outcomes for every individual case. Nonetheless, the standards remain the accepted benchmark against which departments are expected to audit their practice, and persistent breaches, particularly a 0% compliance rate across an entire triage arm, represent a systemic process failure that merits correction irrespective of whether individual patients ultimately experience measurable harm. Prospective outcome data linked to timing would help clarify whether the VFC-associated delays identified here carry functional consequences for this patient group.

Strengths

To our knowledge, this is among the first UK service evaluations to examine the effect of VFC triage specifically on time-to-surgery for distal radius fractures, rather than on time-to-clinic-review, which has been the focus of the majority of published VFC compliance data [7,8,9]. The direct cohort comparison between VFC and traditional trauma-meeting triage, supported by formal statistical testing despite the constraints of a small single-centre sample, allows a specific and testable hypothesis, that of an administrative bottleneck introduced by an additional face-to-face clinic step, to be generated for future prospective, multicentre study.

Limitations

The principal limitation of this evaluation is that it is observational and unadjusted: the association between VFC triage and reduced BOAST compliance cannot be interpreted as causal, and confounding and selection bias remain important alternative explanations that this dataset cannot fully exclude. Allocation to pathway was not randomised, and, as noted in Methods, no standardised or documented protocol governing this allocation could be identified; the specific basis on which individual patients were routed to one pathway rather than the other could not be determined retrospectively. This absence of a defined allocation process is itself a limitation of the pathway as delivered, and it remains plausible that clinicians preferentially recognised patients with more obviously unstable or high-energy injuries as requiring surgery at initial presentation and referred them directly to the trauma meeting, introducing a selection bias whereby the traditional pathway cohort may have included clinically more obvious surgical candidates. Other unmeasured operational factors, including theatre list availability, on-call consultant workload, weekend versus weekday admission, and individual surgeon availability, were not captured as discrete variables and could plausibly differ between the two cohorts, and their potential contribution to the observed timing difference cannot be excluded. Baseline demographic characteristics (age and sex) were not extracted as part of this evaluation, limiting our ability to formally compare the two cohorts for comparability beyond fracture morphology. This evaluation is further limited by its retrospective design, single-centre setting, and small sample size (N = 25), which produced a zero-value cell in the VFC-compliant group and correspondingly wide confidence intervals around the odds ratio. Granular timestamps for each step within the VFC pathway were not prospectively captured, which prevented formal process-mapping of the proposed bottleneck mechanism beyond the clinic-review step itself; this should be addressed in a future audit cycle with structured timestamp collection at each pathway stage. Finally, this evaluation did not assess longer-term functional or patient-reported outcomes, so the clinical consequences of surgical delay for this cohort remain unquantified. Given these limitations, the findings are best regarded as hypothesis-generating rather than definitive evidence of a causal pathway effect, and warrant confirmation through prospective, multicentre evaluation with adjustment for potential confounders.

Recommendations

On the basis of these hypothesis-generating findings, we propose, rather than conclude as evidence-based practice, that departments using a VFC triage model consider a direct-listing escalation protocol for any fracture identified on remote review as meeting BOAST operative criteria, bypassing the additional face-to-face clinic step. We further suggest a re-audit cycle following implementation of any such change to assess its effect, and propose that other NHS trusts using VFC pathways consider auditing their own compliance with BOAST surgical timing standards, given the national reach of the VFC model. These proposals should be regarded as a basis for further prospective evaluation rather than as evidence-based conclusions in their own right.

In practical terms, this could be operationalised through a simple modification to the existing VFC proforma: the reviewing consultant would flag any radiograph meeting BOAST operative criteria for same-day referral directly to the trauma coordinator for theatre listing, rather than routing the patient into the standard outpatient booking queue. Administrative and clinical staff should be made explicitly aware that BOAST-eligible injuries take priority over routine VFC-to-clinic booking. A structured re-audit incorporating timestamp capture at each pathway step, electronic upload, remote review, escalation decision, and theatre date would allow the specific point of delay to be quantified rather than inferred, and would provide an objective measure of whether the escalation protocol succeeds in restoring compliance to a level comparable with, or exceeding, that of the traditional trauma-meeting pathway.

## Conclusions

In this single-centre service evaluation, the VFC triage pathway was associated with significantly lower compliance with BOAST surgical timing standards for distal radius fractures than the traditional trauma-meeting pathway. This association coincided with a documented additional face-to-face clinic step prior to listing in 94% of VFC-pathway patients, but the retrospective, unadjusted design of this evaluation means that the findings should be regarded as hypothesis-generating rather than proof of a causal effect of the VFC pathway itself. While VFC models have demonstrated clear benefits for outpatient efficiency and clinic-review compliance, our findings raise the possibility that they may inadvertently disadvantage the minority of patients who require hyper-acute surgical fixation. Given the high national volume of distal radius fractures and the widespread adoption of VFC triage models across the NHS, this issue, if confirmed, is unlikely to be confined to a single institution. We propose a direct-listing escalation pathway for BOAST-eligible fractures identified on remote review as a candidate intervention for further evaluation, supported by a structured re-audit incorporating stage-level timestamp data to confirm improvement and precisely localise any residual delay within the pathway.
